# Association between ramucirumab-related hypertension and response to treatment in patients with metastatic gastric cancer

**DOI:** 10.18632/oncotarget.24900

**Published:** 2018-04-27

**Authors:** Giandomenico Roviello, Silvia Paola Corona, Andrea Giovanni Multari, Giovanni Paganini, Giorgio Chiriacò, Raffaele Conca, Roberto Petrioli, Daniele Generali, Pietro Rosellini, Michele Aieta

**Affiliations:** ^1^ Division of Medical Oncology, Department of Onco-Hematology, IRCCS-CROB, Referral Cancer Center of Basilicata, 85028 Rionero, Vulture (PZ), Italy; ^2^ Peter MacCallum Cancer Centre, Radiation Oncology Department, Moorabbin Campus, East Bentleigh, Victoria 3165, Australia; ^3^ Unit of Medical Oncology, Department of Oncology, Ospedale San Donato, 52100, Arezzo, Italy; ^4^ Unit of general medicine, Azienda Ospedaliera “C. Poma” Presidio ospedaliero di Pieve di Coriano, ASST Mantova, Italy; ^5^ Medical Oncology Unit, Department of Oncology, ASST del Garda, 25015 Desenzano del Garda (BS), Italy; ^6^ Department of Medicine, Surgery and Neurosciences, Medical Oncology Unit, University of Siena, Viale Bracci - Policlinico “Le Scotte”, 53100, Siena, Italy; ^7^ Department of Medical, Surgery and Health Sciences, University of Trieste, 34129 Trieste, Italy; ^8^ Breast Cancer and Translational Research Unit, ASST Cremona, 26100 Cremona, Italy

**Keywords:** gastric cancer, ramucirumab, hypertension

## Abstract

**Purpose:**

Hypertension (HTN) is frequently associated with the use of angiogenesis inhibitors targeting the vascular endothelial growth factor pathway, such as ramucirumab. The aim of this study was to retrospectively evaluate if occurrence of HTN is correlated with response to second line treatment with ramucirumab+paclitaxel for metastatic gastric cancer.

**Methods:**

Treatment consisted of ramucirumab 8 mg/kg intravenously (iv) on days 1 and 15, plus paclitaxel 80 mg/m^2^ iv on days 1, 8, and 15 of a 28-day cycle. Patients received study treatment until disease progression, unacceptable toxicity, or withdrawal of consent.

**Results:**

Thirty-four patients were retrospectively evaluated. Among these, 6 (17.6%) developed grade 3 ramucirumab-induced HTN. These patients had a better outcome than those with lesser grades events, with a progression-free survival (PFS) of 7.8 months (95% CI 4.4-not reached) versus 4.2 months (95% CI 3.1-5.2) (p=0.001). overall survival (OS) was 11.9 months (95% CI 9.3-not reached) in the grade 3 HTN group, versus 7.2 months (95% CI 5.9-10.1).

**Conclusions:**

Despite the small number of patients and the retrospective nature of the data, our analysis showed that occurrence of ramucirumab-related HTN, in particular G3 HTN, predicts response to treatment with ramucirumab+paclitaxel in patients with metastatic gastric cancer.

## INTRODUCTION

Gastric cancer is considered one of the main causes of cancer-related death worldwide [1, 2]. Unfortunately most patients present with metastatic disease and are candidate to palliative chemotherapy, with very poor outcome. In fact, median overall survival (OS) in these cases is limited to 12 months [3, 4]. Recently, ramucirumab, a novel anti-angiogenic agent has been approved, initially as monotherapy, and subsequently in combination with paclitaxel for second line treatment of patients with metastatic gastric cancer, in the presence of a good performance status [5–8]. Ramucirumab is a human IgG1 monoclonal antibody against the Vascular Endothelial Growth Factor Receptor 2 (VEGFR-2) which prevents ligand binding and receptor-mediated pathway activation in endothelial cells [9]. As expected from an anti-angiogenic agent, hypertension represents a frequent adverse event recorded during treatment with ramucirumab. Recently, two large meta-analyses quantified the risk of occurrence of any grade and high grade (grade 3 and above) hypertension in patients treated with ramucirumab [10, 11]. In the phase III RAINBOW trial, HTN of any grade was reported in 25% of patient treated with the combination of paclitaxel and ramucirumab, while grade 3 HTN occurred in 15% of patients. No grade 4 HTN was reported.

The mechanisms underlying the occurrence of ramucirumab-related HTN are not completely clear. However it has been hypothesized that ramucirumab-mediated inhibition of VEGFR-2 could inhibit several pathways, including phosphoinositide 3-kinase and Akt, as well as reduce the expression of endothelium-derived nitric oxide synthase, leading to decrease in nitric oxide levels with consequent vasoconstriction and decrease in sodium renal excretion. These metabolic changes would ultimately result in development of HTN [12–14].

Unfortunately, less than 30% of patients respond to ramucirumab, this fact underlying the need to identify predictors of treatment efficacy. We performed a retrospective analysis to evaluate whether development of HTN in patients with metastatic gastric cancer receiving ramucirumab is associated with the antitumor effect of the drug.

## RESULTS

### Patient characteristics

From October 2015 to November 2017, a total of 34 patients were enrolled in the study. Baseline patient characteristics are summarised in Table 1. The majority of patients were males (24; 70.6%), with a median age of 64 years (range 39–75). In total, 14 (41.2%) patients had an ECOG performance status of 0. 14 patients (41.1%) received prior surgery, 11 (32.3%) had >2 sites of metastasis and 13 (38.2%) presented peritoneal metastases.

**Table 1:** Patient characteristics

No. of patients	34
**Age, years**	
Median	64
Range	39-75
**Sex**	
Male	24
Female	10
**ECOG PS**	
0	14
1	20
**Tumor location**	
Stomach	26
Gastroesophageal junction	8
**Differentiation**	
Well differentiated	3
Moderate	11
Poorly differentiated	20
**Primary tumor resected**	
Yes	14
No	20
**Previous treatment**	
Triplet	8
Doublet	24
HER2	2
**Time to progressive disease on first-line therapy**	
<6 months	20
≥6 months	14
**Number of metastatic sites**	
0–2	23
≥3	11
Peritoneal metastases	13

Median PFS was 4.5 months (95% CI 3.2-6.2) and median OS was 9.3 months (95% CI 6.8-11), no CR was observed, DCR was 76.5% (26/34 patients) (Table 2).

**Table 2:** Best response according HNT grade

	All patients (n=34)	G0 (n=25)	G1 (n=1)	G2 (n=2)	G3 (n=6)
PR	9	7	0	0	2
SD	17	10	1	2	4
PR + SD	26	17	1	2	6
PD	6	6	0	0	0
NE	2	2	0	0	0
PFS (months)	4.5	4.5	NE	2.2	7.8
OS (months)	9.3	7.2	NE	3.1	11.9

Abbreviations: progression free survival (PFS); overall survival (OS), partial response (PR), stable disease (SD), progression disease (PD), not evaluable (NE)

### Hypertension and clinical outcome

Thirteen patients (38.2%) presented a previous diagnosis of HTN managed with medical treatment. All evaluated patients had normal range blood pressure at baseline. Nine patients (26.5%) developed HTN during treatment (1 patient (2.9%) grade 1, 2 patients (5.9%) grade 2 and 6 patients (17.6%) grade 3, no grade 4 was reported). Six patients (17.6%) started treatment with anti-hypertensive therapy, but no patient discontinued ramucirumab as consequence of HNT occurrence.

**Figure 1:**
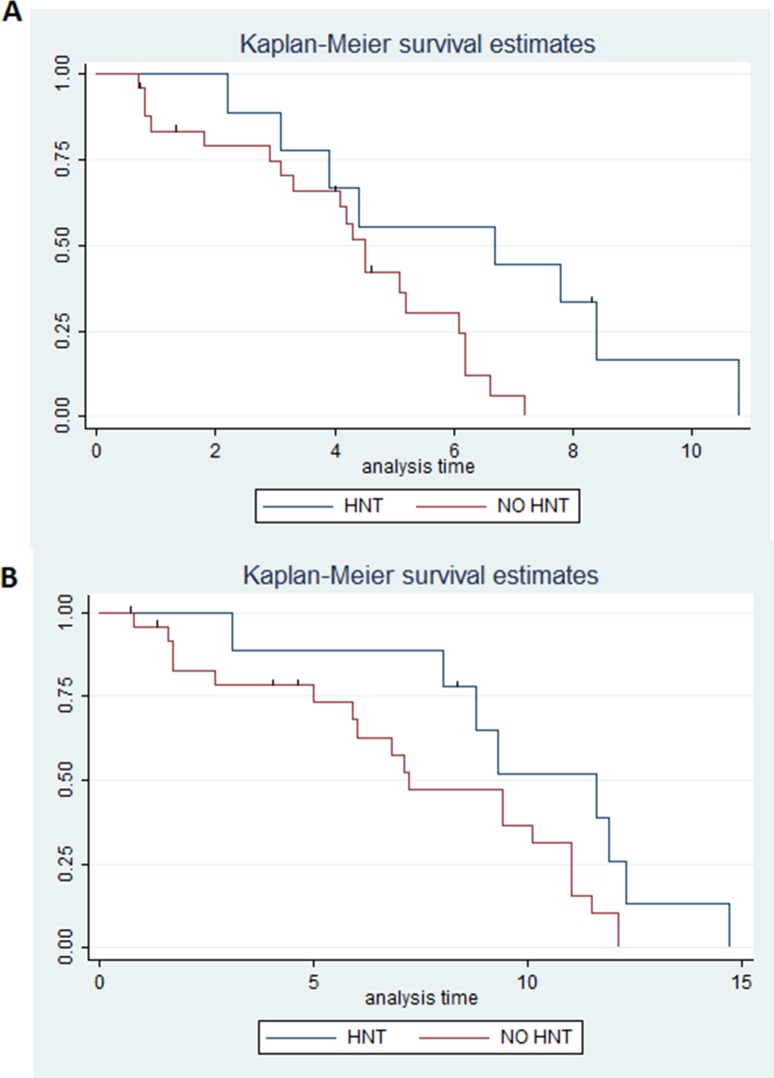
**(A)** Estimated PFS for ramucirumab+paclitaxel in patients with hypertension (blue) or without (red); **(B)** Estimated OS for ramucirumab+paclitaxel in patients with hypertension (blue) or without (red).

Patients who developed HTN had a median PFS of 6.7 months (95% CI 2.2-8.4) in comparison to 4.5 months (95% CI 3.1-6.1) for patients with normal blood pressure (p=0.02) (Figure 1A). HTN patients had a median OS of 11.6 months (95% CI 3.1-12.3) compared to 7.2 months (95% CI 5-11) for those in the non HTN group (p=0.06) (Figure 1B). DCR in HTN patients was 100% compared to 65.4% in those without HTN (p=0.06) (Table 2).

**Figure 2:**
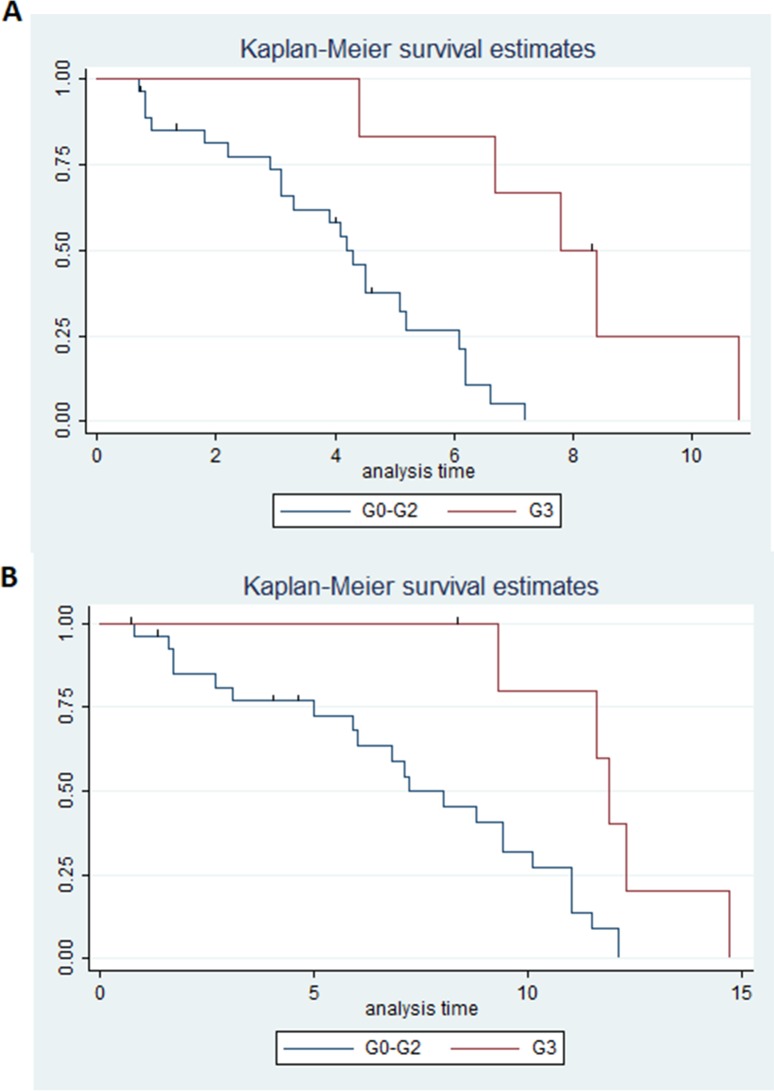
**(A)** Estimated PFS for ramucirumab+paclitaxel in patients with hypertension grade 3 (blue) or hypertension <3 (red); **(B)** Estimated OS for ramucirumab+paclitaxel in patients with hypertension grade 3 (blue) or hypertension <3 (red).

In particular the benefit was superior in grade 3 ramucirumab-induced HTN in comparison to all the other patients, as median PFS was 7.8 months (95% CI 4.4-not reached) in these patients versus 4.2 months (95% CI 3.1-5.2) in all other patients (p=0.001) (Figure 2A). Median OS was 11.9 months (95% CI 9.3-not reached) in patients with grade 3 ramucirumab-induced HTN versus 7.2 months (95% CI 5.9-10.1) in all other patients (p=0.007) (Figure 2B). DCR was 100% in patients with grade 3 drug-induced HTN versus 76.9% in all other patients (p=0.17) (Table 2).

Finally Supplementary Figure 1A and 1B reported Kaplan-mayer of PFS and OS according any grade of HNT.

After adjusting for clinical covariates (peritoneal metastases, ECOG PS, number of metastatic sites, presence of a primary tumour, time to progression since prior therapy, tumour differentiation grade), ramucirumab-related any-grade HNT showed an hazard ratio (HR) of 0.34 for PFS (HR=0.34, 95% CI 0.13-0.37, p=0.02) and of 0.49 for OS (HR=0.49, 95% CI 0.22-1.11, p=0.09). Grade 3 HNT showed a HR of 0.21 for PFS (HR=0.21, 95% CI 0.12-0.42, p=0.002) and of 0.26 for OS (HR=0.26, 95% CI 0.19-0.76, p=0.01), confirming that grade 3 HNT is an independent prognostic factor for response to treatment.

## DISCUSSION

Angiogensis is still considered one of the main actionable targets of anticancer therapies. HTN is a common “on-target” adverse event of the treatment with antiangiogenic drugs and may represent a possible clinical predictive marker of treatment efficacy.

Ramucirumab is an antiangiogenic monoclonal antibody specific for the VEGFR-2. Whilst the occurrence of HTN has rarely led to discontinuation of treatment [11], a recent meta-analyses defined the risk of ramucirumab-related HNT [11], in particular the 9% of patients experienced grade 3 HNT with a relative risk:3.7. In 2017, a meta- with the anti-VEGF bevacizumab reported an HNT incidence of 9.3% and a relative risk: 4.89 [15], while for the “VEGF-trap” aflibercept, another meta-analysis patients reported a summary incidences of high-grade HNT of 17.4 % (OR 4.97) [16]. From these data it seems that ramucirumab may induce lesser HTN than other anti-VEGF compounds.

The mechanisms behind the development of HTN from ramucirumab are similar to those described for other antiangiogenic agents and include: decreased production of nitric oxide in the wall of arterioles and other resistance vessels [17], increased activation of the endothelin-1 system [18], and/or capillary rarefaction [19]. However, ramucirumab blocks angiogenesis by a different mechanism of action compared with other anti-angiogenetis agents, the unique receptor-binding activity of ramucirumab on VEGFR2, that is different respect ligand-binding or “the VEGF-trap”, may explain the diversity of HNT incidence from ramucirumab compared with bevacizumab or aflibercept.

Cumulative data show that bevacizumab-induced HTN appears to be associated with its efficacy. Scartozzi et al evaluated 39 patients with metastatic colorectal cancer treated with bevacizumab as part of front-line therapy [20] showing an in increase in RR and PFS observed in the group of patients who developed bevacizumab-related HTN, however, no statistically significant difference was observed in OS. Österlund et al showed that HTN predicted bevacizumab treatment efficacy and OS [21]. Recently, HTN grade 2-3 was predictive of response to treatment with bevacizumab in metastatic colorectal cancer but not of OS [22]. Finally, similar studies were published for patients with breast, lung and renal cancer confirming the role of bevacizumab-HNT as a predictive biomarker [23–25]. Unfortunately, little data are available about HNT and ramucirumab efficacy in metastatic gastric cancer. In this retrospective study, a correlation emerged between the development of HTN during treatment with ramucirumab and the survival outcomes of patients with metastatic gastric cancer. Patients who developed HTN had a better PFS, RR and OS than those without HTN.

Of note, patients with grade 3 ramucirumab-related HTN showed a significantly higher benefit and better outcomes in comparison to any other patients (Figure 2). In this context, a more recent pharmacokinetic analysis [26] of both REGARD and RAINBOW phase III trials, aimed at evaluating exposure-efficacy and exposure-safety relationships of ramucirumab, showed that higher ramucirumab exposure was associated with longer OS and PFS. Specifically, in the 321 patients treated with ramucirumab+paclitaxel on the RAINBOW study, grade 3 and above HNT and other adverse events such as leukopenia and neutropenia significantly correlated with drug steady state, with increased exposure leading to increased incidence of these side effects. In line with these data, our grade 3 HNT patients showed a better outcome compared to patients with HNT of lesser grades or no HNT. Unfortunately, the small sample size did not allow further analysis and stratification by grades and more studies are awaited to evaluate the significance of lower grade on target adverse events. Furthermore, whilst another exploratory analysis of efficacy and safety of ramucirumab conducted in East Asian patients from the RAINBOW trial [27] confirmed the positive relationship between efficacy and ramucirumab exposure, patients with higher ramucirumab steady state had higher incidence of grade ≥3 leukopenia and neutropenia, but not HNT. This last data may require a specific analysis in prospective studies, taking into account the genetic differences between East Asian and European patients.

It is well known that HNT from antiangiogenic agents is readily managed with anti-hypertension medications [11] and in the near future further studies will be investigating the potential impact of hypertension therapies on treatment efficacy in metastatic gastric cancer patients treated with ramucirumab.

The present study has several limitations such as 1) the retrospective nature of the data, 2) the small number of patients evaluated and 3) the absence of a control arm (patients not receiving ramucirumab). Therefore, it is difficult to draw definitive conclusions and the clinical characteristics of patients may also impact on the findings especially for the multivariate analysis. Nonetheless we observed a strong correlation between occurrence of higher grade HTN and response to therapy with ramucirumab.

In conclusion, ramucirumab-induced HTN (mainly grade 3) is an independent predictor of treatment efficacy and survival in patients with metastatic gastric cancer treated with the combination of ramucirumab+paclitaxel. Prospective large-scale trials are needed to further confirm these results and examine the significance of on target hypertension as a predictive marker of response to therapy.

## PATIENTS AND METHODS

### Eligibility criteria

The study retrospectively evaluated patients with histologically proven advanced gastric cancer, who had documented objective radiological or clinical disease progression during or within 4 months from the last dose of first-line platinum and fluoropyrimidine doublet, with or without anthracycline. Patients eligibility criteria included 18 years of age or more, Eastern Cooperative Oncology Group performance status of 0-1, bi-dimensionally measurable disease, a life expectancy of at least 3 months, adequate haematological parameters (an absolute neutrophil count ≥ 1.5 × 109/l and a platelet count ≥ 100 × 109/l), creatinine and total bilirubin levels <1.25 times the upper normal limit, aspartate and alanine aminotransferase <3.0 times the upper normal limit and absence of a second primary tumour other than non-melanoma skin cancer or in-situ cervical carcinoma. Patients with operable metastatic disease were excluded from the study, as were those with severe cardiac dysfunction, chronic diarrhoea or sites of uncontrolled infection and patients with a history of gastrointestinal perforation, fistulae, arterial thromboembolic event within the previous 6 months, significant gastro-intestinal bleeding, any significant venous thromboembolism (within 3 months before enrolment) or poorly controlled hypertension defined as systolic blood pressure ≥ 160 mmHg or diastolic blood pressure ≥ 95 mmHg. This study was approved by the local ethical and scientific committee and all patients gave their written informed consent.

### Patient evaluation

The pre-treatment evaluation, performed within 2 weeks before study entry, included a detailed history and physical examination, a complete blood cell count with differential and platelet counts, whole-blood chemistry and computed tomography (CT) scans, and/or magnetic resonance imaging (MRI) of the chest and abdomen. Blood pressure measurements were recorded before the infusion of ramucirumab and a daily record of the blood pressure values was kept by the patient. The highest value of arterial blood pressure recorded was taken into account to define the grade of ramucirumab-induced arterial HTN according to the grading of the National Cancer Institute—Common Toxicity Criteria toxicity scale V.4.2 [28]. Treatment response by means of CT scan and/or MRI was evaluated every 3 cycles or sooner if clinically indicated. Tumour response was assessed using RECIST 1.1 criteria [29].

### Treatment delivery

Treatment consisted of ramucirumab 8 mg/kg intravenously (i.v.) on days 1 and 15, plus paclitaxel 80 mg/m^2^ i.v. on days 1, 8 and 15 of a 28-day cycle. Patients received study treatment until disease progression, unacceptable toxicity or consent withdrawal.

### Statistical considerations

This is a retrospective cohort study aiming to investigate the efficacy of ramucirumab in patients who developed on-target HTN in comparison to patients without HTN. Primary end-point was progression free survival (PFS), calculated as the time from the first ramucirumab infusion to disease progression or death. Secondary end points included: overall survival (OS), measured from the date of treatment start to the date of death, and disease control rate (DCR), expressed as the proportion of patients who achieved complete response (CR), partial response (PR) and stable disease (SD) according RECIST criteria. Mean, median, standard deviation and minimum and maximum values were reported for continuous variables, while count and proportion were reported for non-continuous variables. Kaplan-Meier method was used to determine PFS and OS. Long-rank test was performed to analyse PFS and OS in relation to the development of HTN. Comparisons of PFS and OS between groups were performed using Cox Regression. Data on patients who were lost to follow-up were censored at the time of the last evaluation. After univariate analysis, a multivariable Cox regression model (including peritoneal metastases, ECOG PS, number of metastatic sites, presence of a primary tumour, time to progression since prior therapy, tumour differentiation grade) was used to adjust for these potentially confounding factors. The threshold for statistical significance was established at P<0.05. Statistical analysis was performed using STATA software.

## SUPPLEMENTARY MATERIALS FIGURE


