# Supplementary material for: Association between ramucirumab-related hypertension and response to treatment in patients with metastatic gastric cancer
Source: Oncotarget. 2018 Apr 27;9(32):22332–9. doi: 10.18632/oncotarget.24900 (PMC5976467; doi:10.18632/oncotarget.24900)
Supplement: Supplementary file 1 [file oncotarget-09-22332-s001.pdf]

Association between ramucirumab-related hypertension and response to treatment in patients with metastatic gastric cancer

SUPPLEMENTARY MATERIALS

A

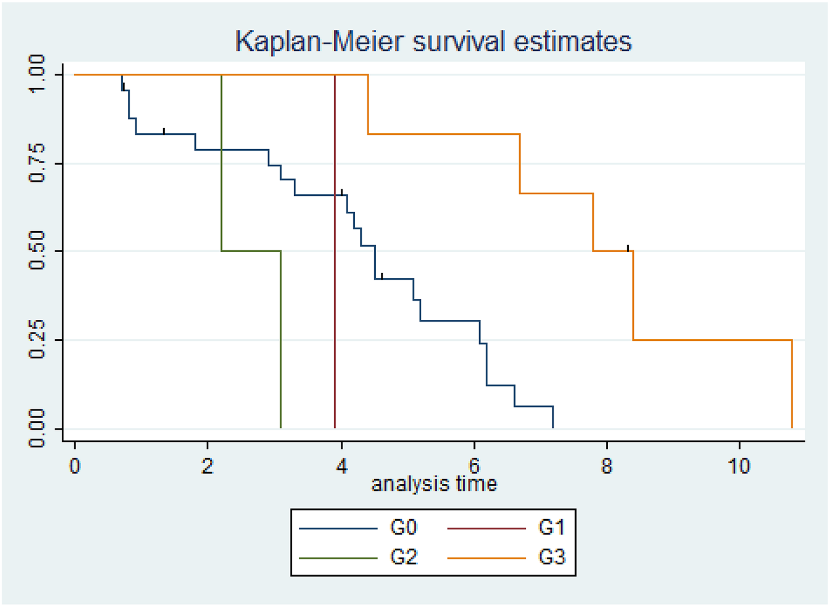

B

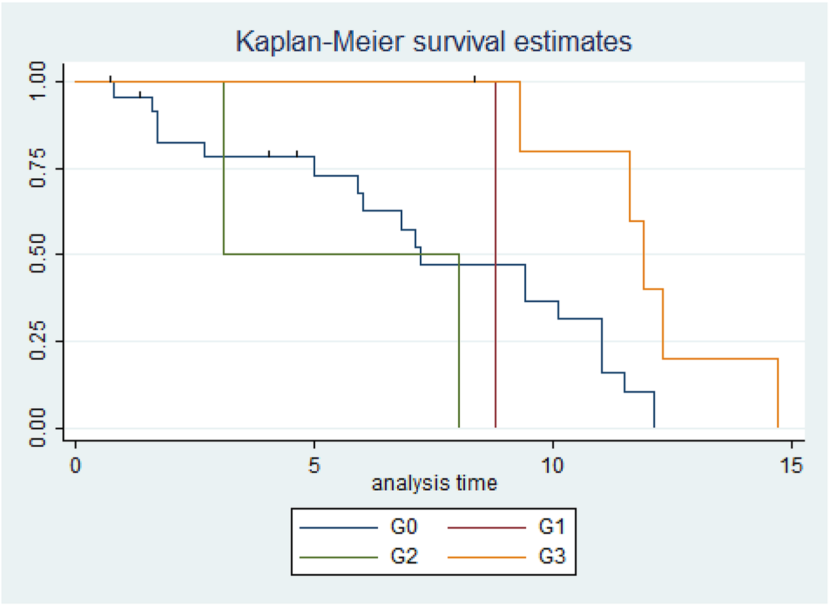

Supplementary Figure 1: (A) Estimated PFS for ramucirumab+paclitaxel according hypertension grade; **(B)** Estimated OS for ramucirumab+paclitaxel according hypertnension grade.
